# Sellar-Parasellar and Petrous Bone Metastasis from Differentiated Thyroid Carcinoma: Imaging Characteristics and Follow-Up Profile Post Radioiodine Therapy

**DOI:** 10.1055/s-0043-1768664

**Published:** 2023-04-28

**Authors:** Sunita Nitin Sonavane, Trupti Upadhye, Sandip Basu

**Affiliations:** 1 Radiation Medicine Centre, Bhabha Atomic Research Centre, Tata Memorial Hospital Annexe, Parel, Mumbai, Maharashtra, India; 2 Homi Bhabha National Institute, Mumbai, Maharashtra, India

**Keywords:** sella, petrous apex, differentiated thyroid cancer, FDG, radioiodine, planar, SPECT/CT, PET/CT

## Abstract

Sella turcica and petrous bone metastasis from differentiated thyroid carcinoma are rare clinical entities, with only a few limited cases reported to date. Two cases, one of sella turcica metastasis and the other of petrous bone metastasis from carcinoma of the thyroid gland, are presented. The cases diagnosed to have arisen from poorly differentiated thyroid carcinoma and follicular carcinoma of thyroid, respectively, subsequently underwent total thyroidectomy, radioiodine (RAI) scans and RAI therapies with iodine-131, external radiotherapy, and levothyroxine suppression with follow-up. Their clinical symptoms gradually subsided, with reduction in serum thyroglobulin, and finally resulted in disease stabilization. With the multimodality therapeutic approach, both patients are alive to date, with 48- and 60-month survival post diagnosis, respectively.

## Introduction

The differential diagnosis of the lesions in the sellar/parasellar region is challenging and is usually clinched through clinical, imaging, histopathological report, and immunohistochemistry (IHC). 1 Sellar and para-sellar metastatic lesions are relatively rare. 2 Well-differentiated carcinoma of the thyroid most commonly presents as nodular thyroid and metastatic disease outside of the neck to other organs is observed in only 1 to 4% of cases, with lungs and skeleton being the most frequent sites of involvement. Thyroid cancer metastasizing intracranial skull bones has been uncommon to encounter.

A case of metastasis to the sella turcica and one case of petrous bone metastasis from a follicular carcinoma of the thyroid gland are presented. Both cases were diagnosed of having thyroid malignancy, subsequently underwent total thyroidectomy, radioiodine (RAI) scans, and oral RAI therapies with ^131^ iodine (I-131), after which their clinical symptoms gradually subsided and resulted in lesion stabilization.

## Case 1

**Fig. 1:**
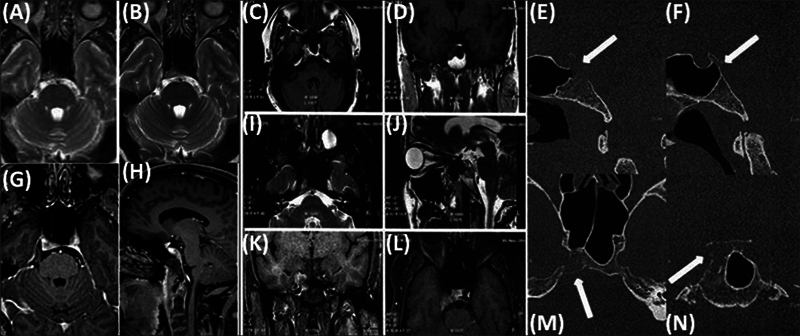
Post-gadolinium fat-saturated T1-weighted brain magnetic resonance images, axial ( **A, B** , **C** , **G** , **I** , **L** , zoomed **M** , **N** ), coronal ( **D** , **K** ), sagittal ( **H** , **J** , zoomed **E** and **F** ), demonstrating enhancing metastatic lytic lesion in the right half of clivus extending anteriorly to involve posterior clinoid and dorsum sella ( *arrow* ).

**Fig. 2:**
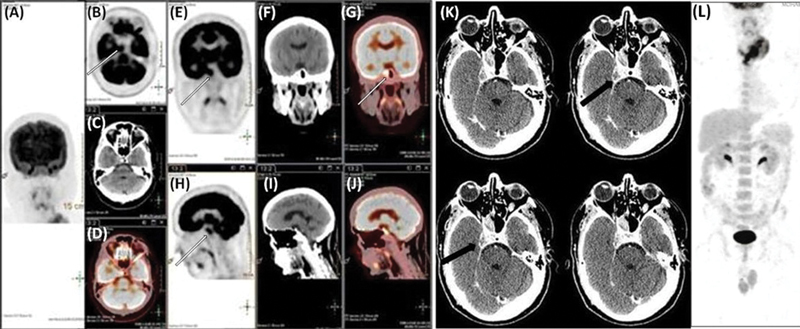
( **A** ) Anterior maximal intensity projection regional head fluorodeoxyglucose-positron emission tomography (FDG-PET); ( **B–D** ) PET, computed tomography (CT), and fused PET-CT axial images; ( **E–G** ) PET, CT, and fused coronal PET-CT; ( **H–J** ) PET, CT, and fused sagittal PET-CT images show metabolically active ill-defined hypodense soft-tissue attenuation mass lesion in right sellar and para-sellar region, maximum standard uptake value (SUV _max_ ) 7.8 ( *solid white arrow* ). Thin slice CT images ( **K** ) delineate hypodense soft tissue mass lesion in the right sellar and para-sellar region ( *solid black arrow* ). ( **L** ) Whole-body FDG PET-CT maximum intensity projection anterior image reveals hypermetabolic soft tissue mass involving thyroid (SUV _max_ : 3.6), few hypermetabolic left cervical level II nodes (SUV _max_ : 6.6).

**Fig. 3:**
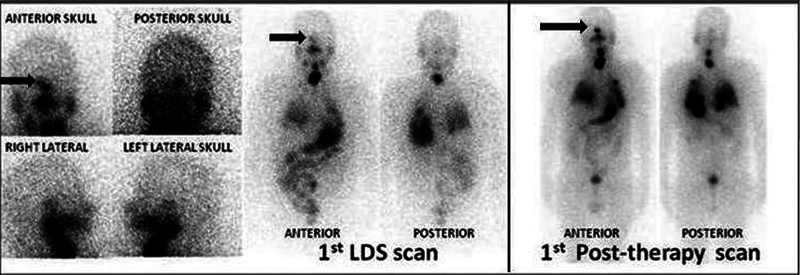
Whole body and skull static images of first iodine-131 (I-131) diagnostic and post-therapy scan (after I-131 therapy) showing radioiodine avid right sella lesion ( *solid black arrow* ), neck remnant, and heterogenous bilateral lung parenchymal uptake.

**Fig 4:**
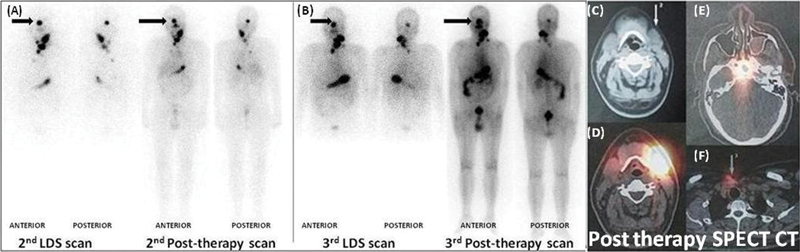
(A) Whole-body scans of second ( **A** ) and ( **B** ) third iodine-131 diagnostic and post-therapy scan (after 245mCi) showing radioiodine avid right sella lesion ( *solid black arrow* ), neck remnant, multiple left cervical nodes, and heterogenous mild chest uptake. Post-therapy head and neck single-photon emission computed tomography-computed tomography (SPECT-CT) images ( **C** – **F** ) reveal iodine avid lesions: axial neck images ( **C** , **D** ) showing left level II nodes, axial images at the level of sella ( **E** ) right sella residual lesion, axial images supraclavicular plane ( **F** ) residual neck remnant.

A 39-year-old male patient presented with diplopia and neck swelling, thyroid fine-needle aspiration cytology (FNAC) revealed follicular neoplasm, magnetic resonance imaging brain revealed enhancing lytic metastatic lesion in the right half of clivus extending anteriorly to involve posterior clinoid and dorsum sella ( Fig. 1 ); further ^18^ F-fluorodeoxyglucose (FDG) photon emission tomography/computed tomography (PET/CT) neck and whole-body images revealed an ill-defined hypodense soft tissue mass lesion noted in the right sellar and para-sellar region, with maximum standardized uptake value (SUV _max_ ) of 7.8 ( Fig. 2 ). Increased metabolic activity was also noted in soft tissue lesion involving the thyroid, SUV _max_ 3.6, a few hypermetabolic left cervical level II nodes (SUV _max_ : 6.6), and non-FDG avid tiny left lung nodules. The patient underwent external radiotherapy to the right sellar lesion with 39Gy/13# under steroid cover. Post-procedure, the patient complained of persistent blurring of distant vision; clinically the patient had persistent left lower rectus palsy, and no other cranial nerve palsies. Thus, the patient diagnosed with solitary sella turcica (bone) with lung and nodal metastasis underwent total thyroidectomy with bilateral central compartment clearance and neck dissection. Histopathology of total thyroidectomy specimen revealed poorly diﬀerentiated thyroid carcinoma with vascular and lymphatic invasion and extrathyroidal extension associated with inﬁltration of perithyroidal fat and skeletal muscle. Post-total thyroidectomy, he received three courses of I-131 therapy (cumulative dose of 701mCi) following thyroxin withdrawal ( Figs. 3 and 4A, B ). The I-131 post-therapy single-photon emission computed tomography/computed tomography (SPECT-CT) images of skull region revealed iodine avid right sella residual lesion ( Fig. 4C–F ). There was classical history given by the patient that on thyroxin withdrawal there was the aggravation of right convergent squint that improved post-I-131 therapies. The stimulated thyroglobulin (Tg) before last RAI therapy was 1.18 ng/mL and follow-up on thyroid hormone suppression therapy was instituted. The patient is clinically having stable disease after adjunctive therapy with levothyroxine suppression and on regular follow-up showing stable sella lesion and no tumor recurrence at 48 months after the primary surgery.

## Case 2

**Fig. 5:**
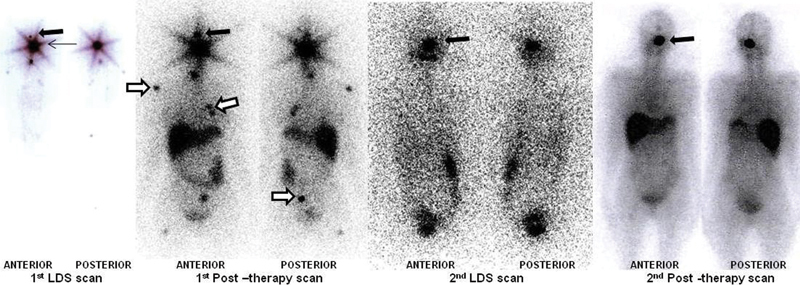
The post-surgery first iodine-131 diagnostic and first post-therapy whole body scan (after 243mCi) showing radioiodine avid petrous temporal region ( *solid black arrow* ), neck residual ( *black line arrow* ) with other bone metastasis ( *solid white arrow with black border* ) at the upper end of right humerus, ribs, left pelvis, and right femur. The second iodine-131 diagnostic and second post-therapy whole-body scan (after 247mCi) showed radioiodine avidity in the left temporal region only ( *solid black arrow* ).

**Fig. 6:**
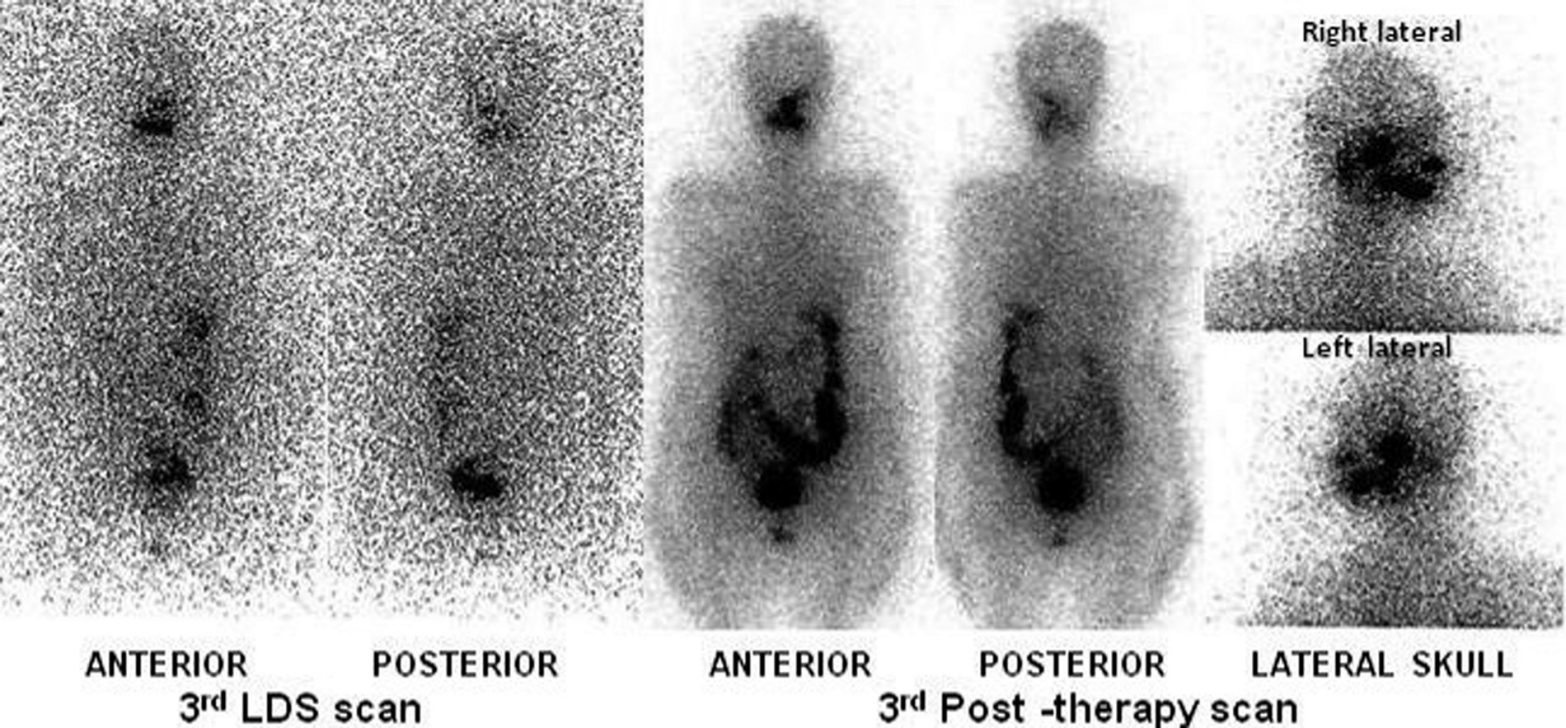
The third iodine-131 diagnostic and post-therapy whole-body scan and skull lateral static images (after 259 mCi) showing radioiodine avidity in the left temporal region only.

**Fig. 7:**
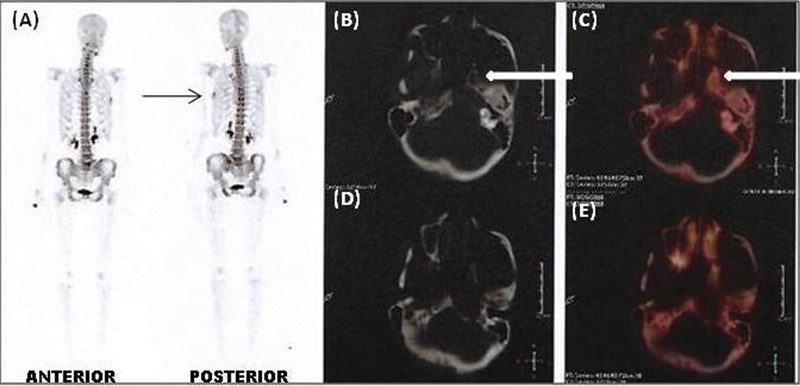
( **A** ) Whole-body ^18^ F-fluoride positron emission tomography (PET) anterior and posterior maximum intensity projection images revealed focus of increased tracer uptake in the left 5th rib laterally ( *black line arrow* ), maximum standard uptake value 5.9. Axial computed tomographic (CT) images ( **B** , **D** ) reveal abnormal well-defined lytic lesion involving the medial half of the petrous part of the left temporal bone with associated soft tissue mass measuring 2.6 × 2.2 cm. Axial PET-CT fusion images ( **C** , **E** ) show no definite ^18^ F-fluoride uptake involving the medial half of the petrous part of the left temporal bone.

A 46-year-old female patient presented with decreased sensation on the left side of the face, diplopia, and headache for 4 months, while on examination fundus showed early papilloedema, and cranial nerve examination elicited depressed left corneal reflex, decreased sensation left side of the face, and left sixth nerve paresis, while other cranial nerves, motor, sensory, and cerebellum were normal. CT brain showed mass lesion arising from the fifth cranial trigeminal nerve with a middle cranial and posterior cranial fossa component, and the patient underwent left temporal craniotomy by subtemporal approach and microsurgical excision of trigeminal nerve tumor, which intraoperatively revealed a grayish firm highly vascular mass in cavum trigeminal area. There was torrential intraoperative bleeding; the excision had to be stopped and a marker clip put at the site of excision. Histopathology of biopsy mass around trigeminal nerve revealed closely packed follicles and glands lined up by tall columnar and cuboidal cells with eosinophilic to clear cytoplasm confirming differentiated follicular tumor, while on IHC tumor cells were TTF1 positive and in the clinical context, the findings were consistent with deposits of follicular carcinoma of the thyroid. Because of subtotal resection of mass around the trigeminal nerve, the patient received EBRT 54 Gy/30 #. The thyroid gland was evaluated showing suspicious nodules in both lobes, and the FNAC of TIRADS V nodule diagnosed follicular carcinoma, and the patient underwent total thyroidectomy. The final histopathology revealed widely invasive follicular carcinoma of the thyroid, and the IHC revealed tumor cells were TTF1positive. Post thyroidectomy-131 scan revealed intense I-131 uptake in the petrous temporal region, neck residual with cervical and mediastinal nodes and upper end of right humerus, pelvis, right femur ( Fig. 5 ), Tg level was 54.35 ng/mL, and she was administered RAI therapy of 243mCi. The subsequent I-131 scan revealed uptake in the left temporal region only ( Figs. 5 and 6 ). The CT skull revealed soft tissue in the region of the petrous apex. ^18^ F-fluoride PET-CT was performed to correlate that revealed an abnormal well-defined lytic lesion involving the medial half of the petrous part of the left temporal bone with associated soft tissue mass measuring 2.6 × 2.2 cm. No definite fluoride uptake was noted in this region ( Fig. 7 ). There was a focus of increased tracer uptake seen in the left fifth rib laterally (SUV _max_ : 5.9); however, there was no metastatic lesion correlated on CT and I-131 scan. The patient received thyroid replacement and suppressive therapy with levothyroxine and underwent total of three RAI therapies using ^131^ I (cumulative dose: 747mCi). The stimulated serum Tg after three therapies was 1.82 ng/mL with negative antibodies. The patient presently has no clinical symptoms. To date, she is alive without evidence of tumor recurrence or metastasis at 60 months after surgery.

## Discussion

The possible metastatic pathways to the pituitary and parasellar region include direct blood-borne metastasis to the posterior pituitary lobe, pituitary stalk, clivus, dorsum sellae, or cavernous sinus or leptomeningeal spread with involvement of the pituitary capsule. 3 Breast and lung malignancies have been reported to be the most common tumor sites of origin in both sexes, while liver, kidney, colon, and thyroid carcinoma and melanoma are rare sources of distant metastases to this region. The suggestive symptoms include rapid onset of progressive ophthalmoplegia with retro-orbital or facial pain and visual impairment. 2 Simon et al mentioned metastatic lesions should always be in the differential diagnosis of a sellar mass, even in young patients. 4 Sella turcica metastases from thyroid carcinoma are exceedingly rare and currently, there are no specific established therapeutic guidelines. 5

Matsuno et al through their analysis of case reports of skull base metastases from thyroid carcinoma concluded that skull base metastasis is a rare clinical entity and can be the initial clinical presentation of more than half of the reported cases of follicular thyroid carcinoma (FTC) and papillary thyroid carcinoma in the presence of silent primary sites, which emphasize the unpredictable nature of thyroid carcinoma, 6 as seen in our patients.

Johnson and Atkins, on the other hand, described a patient with thyroid carcinoma treated by thyroidectomy who developed a destructive metastasis in the sella turcica 6 years later. 7 Our case patient presented with cranial nerve involvement and sellar lesion. In the case reported by Johnson and Atkins, there was temporary improvement followed by treatment with external radiotherapy, and ablation of the metastasis occurred only after the administration of 100mCi of ^131^ I. 7 In our case of sellar lesion despite external radiotherapy and multiple RAI therapies (total 701mCi), the lesion regressed in volume, but still demonstrated persistent iodine uptake in the ^131^ I scan; however, serum Tg (stimulated) was substantially reduced (< 1.2 ng/mL in the last follow-up).

Matsuno et al mentioned that no histopathological features that could predict bone metastasis, particularly skull base metastasis, of differentiated thyroid carcinoma (DTC) were found. 6 Similarly, no particular histological features that could distinguish between DTC metastasizing to the skull base and the other sites were found 6 and no prognostic difference between DTC with skull base metastasis and those with other bone metastases in the literature review were found. 6 In case one, we noted adequate RAI uptake in metastatic lesions of poorly DTC on diagnostic ^131^ I scan. Thiagarajan et al in their recent study studied 35 treatment naïve poorly DTC patients who underwent surgery followed by RAI ablation, with a cumulative median dose of 220mCi (range: 40–1,140). Sixteen patients received more than one RAI treatment for distant metastases and concluded that whenever sodium iodide symporter expression is present in poorly DTC, RAI therapy will be effective. 8 Also, in case one, the FDG-PET/CT study contributed value to the diagnoses of the metastatic and primary lesions. Kumar and Basu showed FDG positive incidental poorly DTC 9 and Hsieh et al showed increased uptake of ^18^ F-FDG was observed in anaplastic and poorly DTC cells, and PET-positive tumors are more likely to be resistant to ^131^ I treatment. 10

DTC metastasizing to the temporal bone is still rare. Very few case reports of thyroid carcinoma metastasizing to the temporal bone have been reported in the past. A review of the literature reveals that in most case reports, the histological types have not been mentioned. 11 Any patient presenting with facial palsy, sudden sensorineural deafness, and periauricular swelling should raise suspicion of a temporal bone tumor. 12 The petrous apex is the commonest site of involvement in the metastatic tumors of temporal bone. The primary tumor most commonly associated is the breast. Other sites of primary tumors included the thyroid, brain, lungs, prostate, and blood (leukemia). 13 Ota et al compared the efficacies of ^18^ F-fluoride positron emission tomography ( ^18^ F-fluoride PET)/CT, ^18^ F-fludeoxyglucose PET ( ^18^ F-FDG PET)/CT, and ^99m^ Tc-MDP bone scintigraphy [planar and single-photon emission CT (SPECT)] for the detection of bone metastases in patients with DTC and concluded the sensitivity and accuracy of ^18^ F-fluoride PET/CT for the detection of bone metastases of DTC are significantly higher than those of ^99m^ Tc-bases conventional skeletal scintigraphy (planar). However, the sensitivity and accuracy of ^99m^ Tc-based planar bone scintigraphy are improved near to those of ^18^ F-fluoride PET/CT when SPECT is added to the planar scan. The sensitivity of ^18^ F-FDG PET/CT is significantly lower than that of ^18^ F-fluoride PET/CT or ^99m^ Tc-MDP bone scintigraphy (SPECT). 14 Such findings provide evidence for employing ^18^ F-fluoride PET-CT in thyroid carcinoma patients with skeletal metastases; in case two, this provided better anatomical delineation of the petrous temporal bone metastasis arising from FTC.

Shen et al mentioned correct preoperative diagnosis of calvarial metastasis from FTC is difficult because of its rarity, and the patients can survive for years after combined therapy including radical operation of thyroid carcinoma, resection of metastatic tumor, adjunctive therapy of levothyroxine, ^131^ I RAI, and skull radiotherapy. 15 External radiation should be administered to patients with low grade/absent uptake in the lesions identified by ^131^ I scintigraphy, 16 while chronic suppression of endogenous thyroid stimulating hormone should be induced by the administration of thyroid hormone to prevent tumor growth. 17 Both of our patients, post-thyroidectomy, post-RAI therapy, and on levothyroxine suppression, are alive to date with 48- and 60-month survival post-diagnosis, with the aforementioned multimodality therapeutic approach.
